# Treacherous territory: Temple fillers and tissue necrosis

**DOI:** 10.1002/ski2.392

**Published:** 2024-04-25

**Authors:** Rawan Almutairi, Saima Usmani, Sarah Mubarak, Wael Aldaraji

**Affiliations:** ^1^ Dermatology Department Ministry of Health Safat Kuwait; ^2^ Myskin Clinic Kuwait City Kuwait; ^3^ Laser and Skin Clinic Baghdad Iraq; ^4^ The Ghanem Clinic London UK

## Abstract

Temporal region of the face is considered the most promising facial region for inducing panfacial effects, playing an increasingly important role in facial contouring and rejuvenation surgeries, which in turn has led to a rapid growth in the demand for aesthetic correction for temporal hollowing. In order to correct this issue and achieve a more youthful appearance, filler injections can be used. Although this procedure is generally safe and is increasingly popular, complications may happen. Complications include visual loss, neurological deficits, embolism, and non‐thrombotic pulmonary embolism. These complications are thought to result from injection of filler material into facial arteries and veins. We describe here a patient who developed pain in the parieto‐temporal region following hyaluronic acid (HA) injection for temporal augmentation, which was complicated by tissue necrosis and alopecia. This was managed with hyaluronidase injection and aspirin tablets. Patient was followed for 6 months period after which she had complete hair growth and total resolution of skin necrosis without any scar formation. Since complications of HA injection are wide and serious, practitioners should always be aware of potential consequences of such procedures.

## Graphical abstract

This is a case report that highlights complications of hyaluronic acid injections, including skin necrosis and localised alopecia, that is successfully managed using hyaluronidase injection.

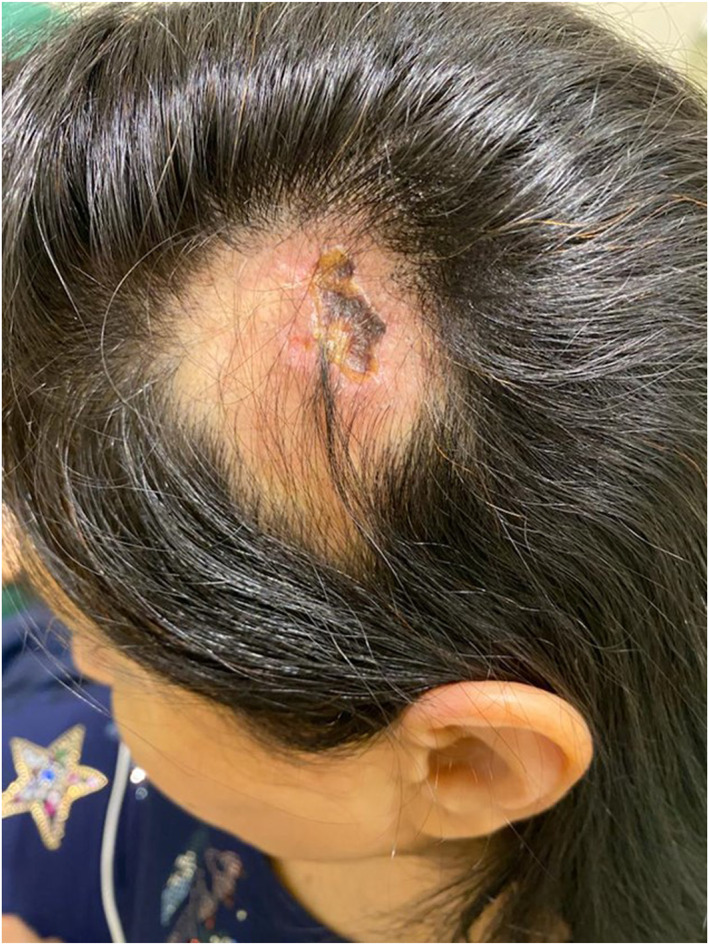

## 1 INTRODUCTION

The body undergoes observable changes with ageing, which include thinning of the epidermis, loss of elasticity, atrophy of the muscle and subcutaneous fat, and bony changes. These changes result in loss of volume, which leads to a gaunt appearance of the temporal region.^1^, ^2^ This region is considered the most promising facial region for inducing panfacial effects, playing an increasingly important role in facial contouring and rejuvenation surgeries, which in turn has lead to a rapid growth in the demand for aesthetic correction for temporal hollowing. In order to correct this issue and achieve a more youthful appearance, filler injections can be used. This procedure is generally safe and is increasingly sought out in order to gain a more aesthetically pleasing look. According to the 2019 annual statistics report of The Aesthetic Society, a total of 749 409 soft tissue filler injections of hyaluronic acid (HA) were perform in the United States. When compared to 2015, an increase by 26.4% is shown, which indicates and growing desire for aesthetic enhancement.^3^ Nonetheless, the procedure is not without complications, especially when done in high‐risk areas, such as the temporal region. Complications include visual loss, neurological deficits, embolism, and non‐thrombotic pulmonary embolism. These complications are thought to result from injection of filler material into facial arteries and veins. Additionally, tissue necrosis due to superficial temporal artery occlusion has recently been reported as a complication of filler injections using temporal lifting technique.^4^ Alopecia is another newly reported complication post‐filler injection surmised to be due to vascular compromise. We describe here a patient who developed pain in the parieto‐temporal region following HA injection for temporal augmentation, which was complicated by tissue necrosis and alopecia, and successfully managed using hyaluronidase (HYAL) injection.

## 2 CASE PRESENTATION

**FIGURE 1:**
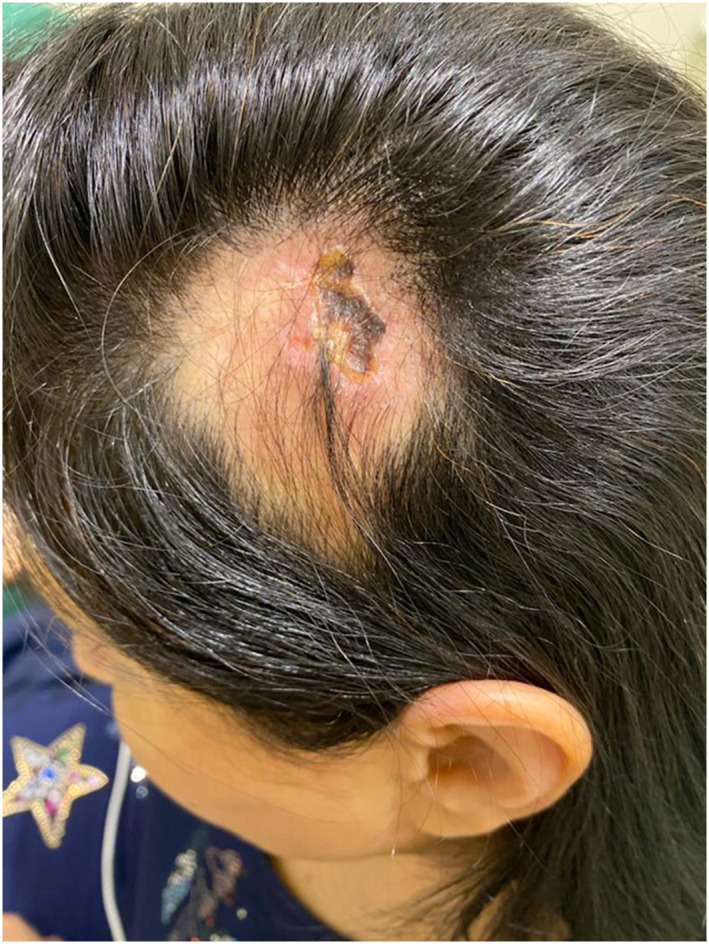
Patch of alopecia and red discolouration in the left parieto‐temporal region measuring approximately 5 cm × 7 cm with ulcerated skin covered by eschar.

**FIGURE 2:**
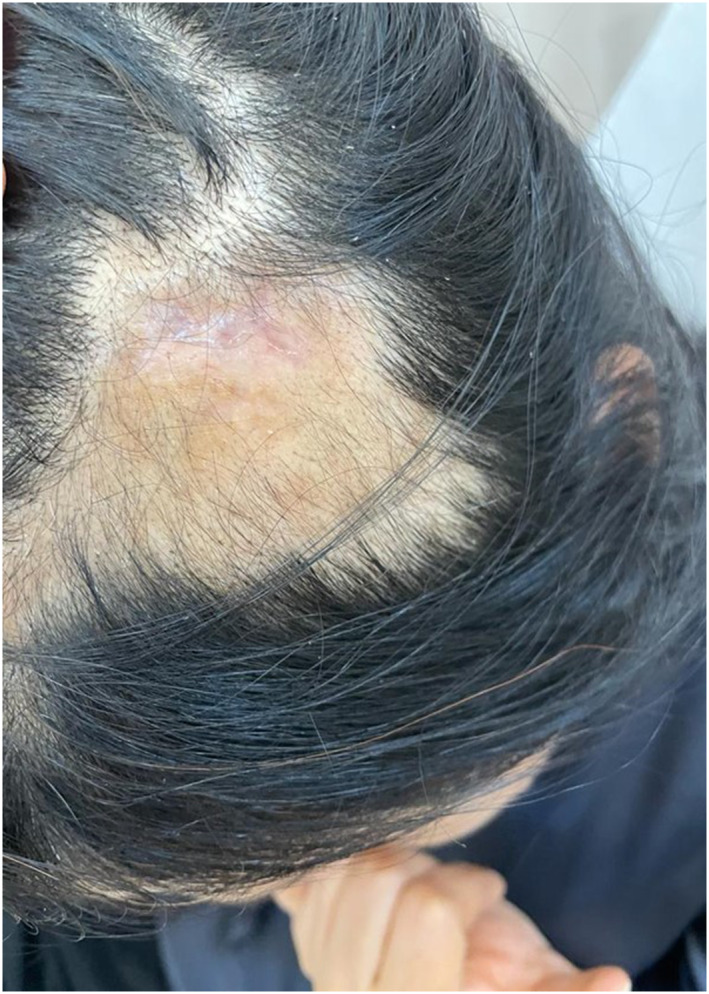
Two weeks follow up showed resolution of the ulcerated lesion with presence of fibrous tissue.

**FIGURE 3:**
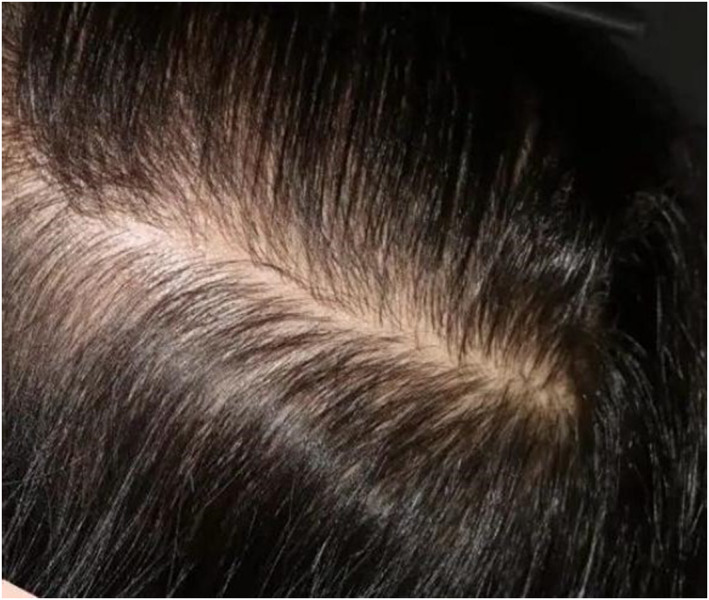
Complete hair growth and total resolution of skin necrosis without any scar formation after 6 months of follow up.

A healthy non‐smoking 48‐year‐old female underwent bilateral temple augmentation using a technique similarly mentioned in Hernandez et al.^5^ Cutaneous access was achieved 1 cm anterior to the apex of the tragus using a 25G needle and a 27G blunt‐tip cannula. The cannula was advanced subdermally in a superior direction proceeding towards the superior posterior temporal region. A 1.0cc bolus of HA‐based soft tissue filler (Restylane Lyft, Galderma) was injected slowly. Two hours after the filler was administered, the patient developed acute onset of severe pain in the left temporal area around the injection site but she did not seek medical advice. Over the course of 10 days after the procedure, the patient noticed progressive hair loss in the same area with a developing central necrotic wound. On day 10 after HA filling, she presented with a patch of alopecia and discolouration in the left parieto‐temporal region measuring approximately 5 cm × 7 cm with ulcerated skin covered by eschar measuring 1.5 cm × 3 cm and reddish discolouration in the immediate surrounding area (Figure 1). Vascular occlusion was suspected and injection of HYAL was administered 200 units subcutaneously in the same area three times half‐hourly. Additionally, aspirin 81 mg once daily was prescribed for 5 days. After 2 weeks, the patient showed resolution of the ulcerated lesion with presence of fibrous tissue (Figure 2). While after 6 months period, she had complete hair growth and total resolution of skin necrosis without any scar formation (Figure 3).

## 3 DISCUSSION

Incidence data related to complications of filler injections is very poor due to underreporting. However, it is widely acknowledged that these complications are occurring at a higher frequency, presumably due to the increasing popularity of said procedure and the growing number of less experienced practitioners performing it.^6^

One of several ways to minimise the risk of vascular occlusion from HA injections is undergoing the procedure under the care of a skilled and experienced practitioner with detailed knowledge of applied anatomy. Methods to prevent complications include aspiration prior to injection to confirm the injection is not intravascular, slow injection technique under low pressure using the smallest volume to achieve the desired effect, and use of blunt‐ended cannulas of 25G or large bore diameters, which are less likely to penetrate vessels. Practitioners should avoid bolus injections in areas at risk of vascular occlusion, avoid adrenaline use as it can mask blanching produced by occlusion, and also avoid areas of previous scarring because deep tissue scars can fix vessels in place and make them easier to penetrate.^6^, ^7^, ^8^, ^9^

If blood supply is suspected to be compromised, the practitioner should immediately discontinue injecting any more product. Conservative measures such as massaging, tapping over, or applying heat to the affected area might possibly resolve the occlusion. However, the mainstay of management is HYAL. Treatment of the ischaemia is accomplished by diffuse injection of HYAL into the affected tissue. It is not necessarily essential to inject directly into the vessel if occluded, as HYAL tends to diffuse widely. Even so, ultrasound guided HYAL injection may be preferred due to accurate localization of filler.^10^ Aspirin is also used as an antiplatelet agent and in order to prevent further clot formation. Antibiotics are suggested in case of necrosis, as the dead cells and tissue are prone to secondary opportunistic infection. Superficial debridement and appropriate wound care may be required to remove the dead tissue, promote healing, and minimise the risk of scarring. However, debridement of vascular occlusion cases should be delayed to perform for reduce the risk of scarring as reported in some consensus paper. Analgesia may be needed in cases of necrosis as it can cause severe pain. Although there is limited evidence, hyperbaric oxygen therapy should be considered in patients with progressive necrosis that is not responding to initial treatment methods. Other treatment options include low molecular weight heparin to prevent thrombosis and embolisation or oral vasodilators to treat vascular occlusion, however evidence to use either treatment on a larger scale is lacking. Nitroglycerin paste has been used in the past to induce vasodilation, yet a recent study by Hwang et al.^11^ suggests no improvement in outcome and worsening perfusion. Immediate and regular follow‐up is of the utmost importance in all cases with complications.^7^, ^12^, ^13^

Although it has been shown that in the literature late application of HYAL more than 24 h after HA injection has not proven effective in avoiding skin necrosis,^14^ in our patient, complete resolution of skin necrosis was achieved even 10 days after injecting HA. Early use of HYAL is always encouraged to manage skin necrosis to achieve reversible outcomes.

## 4 CONCLUSION

We acknowledge that vascular occlusion disrupts the essential blood supply to hair follicles, which plays a role in the abrupt cessation of the anagen phase and the subsequent formation of anagen effluvium. Since complications of HA injection are wide and serious, practitioners should always be aware of potential consequences of such procedures.

## CONFLICT OF INTEREST STATEMENT

The authors declare no conflicts of interest.

## AUTHOR CONTRIBUTIONS

**Rawan Almutairi**: Conceptualization (equal); data curation (equal); formal analysis (equal); funding acquisition (equal); investigation (equal); methodology (equal); project administration (equal); resources (equal); software (equal); supervision (equal); validation (equal); visualization (equal); writing—original draft (equal); writing—review and editing (equal). **Saima Usmani**: Conceptualization (equal); data curation (equal); formal analysis (equal); funding acquisition (equal); investigation (equal); methodology (equal); software (equal); supervision (equal); validation (equal); visualization (equal); writing—original draft (equal). **Sarah Mubarak**: Conceptualization (equal); data curation (equal); formal analysis (equal); funding acquisition (equal); investigation (equal); methodology (equal); project administration (equal); resources (equal); software (equal); supervision (equal); validation (equal); visualization (equal); writing—original draft (equal); writing—review and editing (equal). **Wael Aldaraji**: Conceptualization (equal); data curation (equal); formal analysis (equal); funding acquisition (equal); investigation (equal); methodology (equal); project administration (equal); resources (equal); software (equal); supervision (equal); validation (equal); visualization (equal); writing—original draft (equal); writing—review and editing (equal).

## ETHICS STATEMENT

Not applicable.

## Data Availability

Data sharing is not applicable to this article as no new data were created or analysed in this study.
